# A Loss for Words: A Case of Aphemia

**DOI:** 10.7759/cureus.80165

**Published:** 2025-03-06

**Authors:** Ty J Merry, Sneh Parekh, Vanshika Tripathi, Pramod Reddy

**Affiliations:** 1 Internal Medicine, College of Medicine, University of Florida, Jacksonville, USA

**Keywords:** aphasia, aphemia, cerebral vascular accident, comprehensive physical exam, neuro mri, stroke complications

## Abstract

Aphemia, also known as pure motor mutism, is a rare disorder characterized by the loss of motor function to produce speech while retaining the ability to understand, read, and write language. Differentiating between aphemia and aphasia can be challenging in many cases, as they both present with similar physical exam findings. The key distinctions lie in the location of the insult in the cerebral parenchyma, the ability to understand language, and the associated dysphagia. We present the case of a 78-year-old male who presented with new-onset aphasia and was found to have aphemia based on physical examination and imaging findings.

## Introduction

The etiology of the word "aphemia" derives from Greek: "a-" meaning without and "-pheme" meaning voice, defining a condition as "without voice." This highlights the fact that aphemia is primarily a motor rather than a language disorder, with strokes localized to the motor cortex usually being the culprit. Specifically, previous cases have shown infarcts to the left precentral gyrus and dominant inferior gyrus [1,2]. Injuries to Broca's area, Wernicke's area, and the arcuate fasciculus have been associated with aphasia [3]; however, these areas are less associated with motor control of the muscles of phonation and more predominantly involved with the processing of spoken or written language. While aphasia shares many of the physical findings with aphemia, including lack of speech production, the key difference is that aphasia impairs an individual’s ability to understand and process language. For example, Broca’s aphasia is characterized by non-fluent speech but preserved speech comprehension, while Wernicke’s aphasia is characterized by nonsensical speech that is often meaningless [4]. Aphasia can also manifest itself as paraphasia, difficulties with word findings, and agraphia [5]. Comparatively, aphemia is an injury to the motor cortices, with the primary dysfunction of the production of speech, and patients are largely able to communicate through other means including writing, hand gestures, or facial grimaces [2]. Patients who ultimately suffer from either condition will have challenges communicating their thoughts and needs with others, making it hard to integrate themselves back into daily life. It is important to appropriately diagnose aphemia from aphasia as the therapy is individually tailored for the specific motor or language deficiency, respectively.

## Case presentation

**Figure 1:**
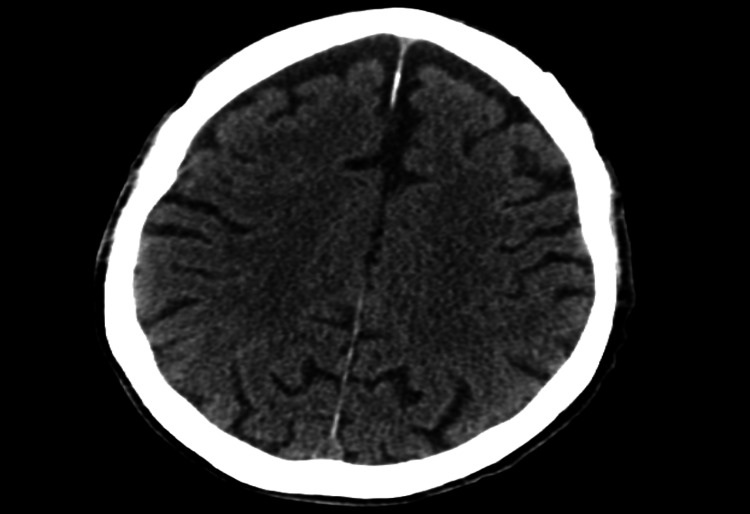
CT head without contrast showing no intracranial hemorrhage or acute infarct, primarily in the left precentral gyrus. CT, computed tomography

**Figure 2:**
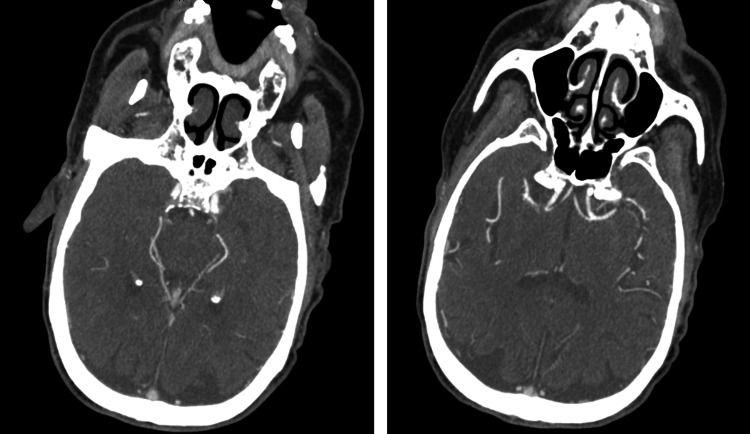
CT angiogram showing a patent Circle of Willis, anterior cerebral artery, and middle cerebral artery. CT, computed tomography

**Figure 3:**
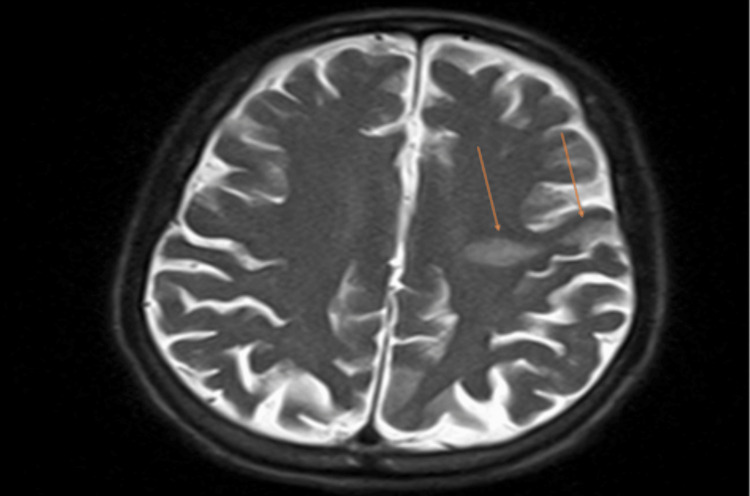
MRI brain without contrast in the T2/FLAIR sequence showing a hyperintense lesion consistent with an infarct in the left precentral gyrus, as indicated by the orange arrows. MRI, magnetic resonance imaging

**Figure 4:**
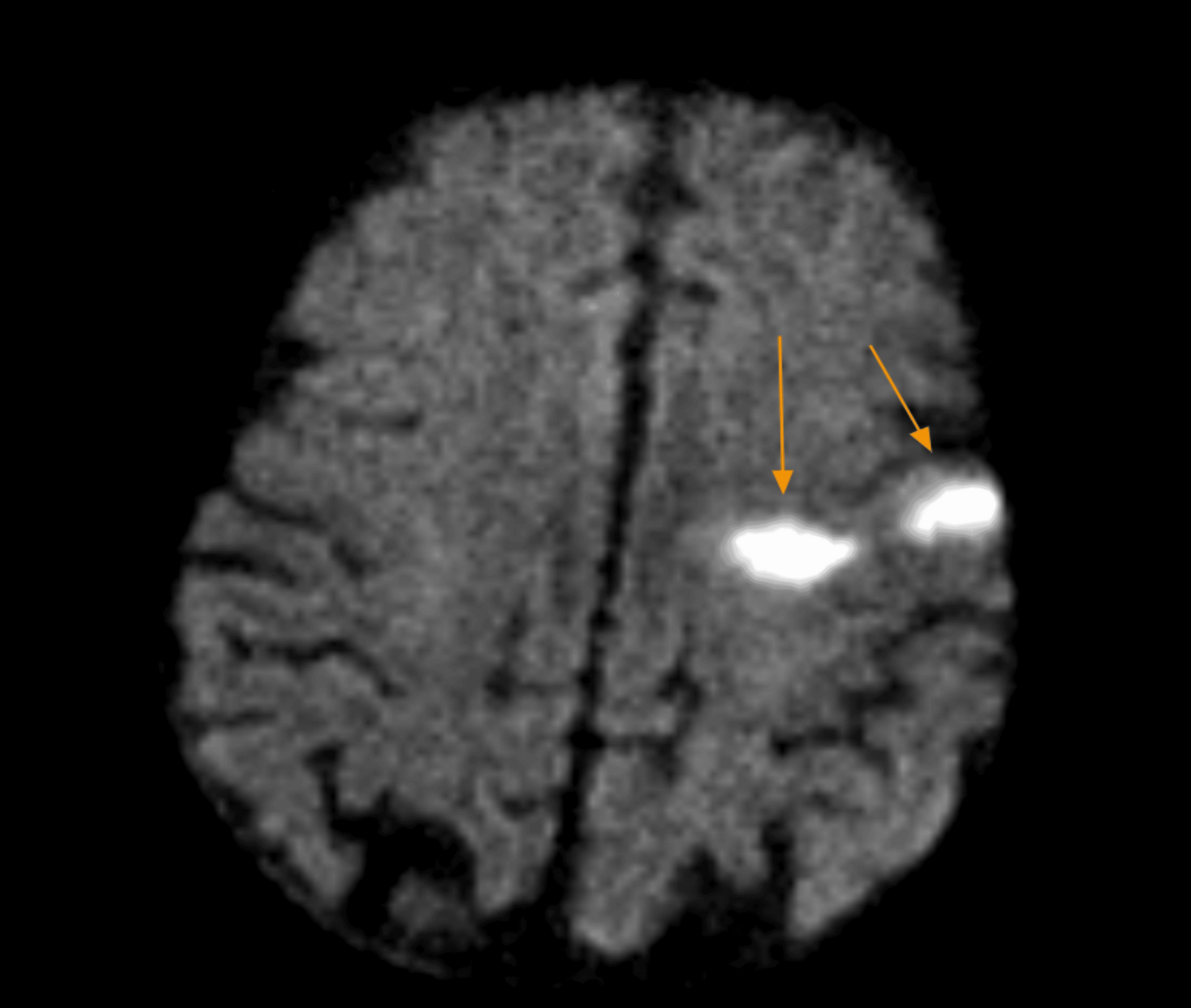
Diffusion-weighted imaging of the MRI brain showing a hyperintense lesion consistent with an infarct in the left precentral gyrus, as indicated by the orange arrows. MRI, magnetic resonance imaging

A 78-year-old male with a past medical history of coronary artery disease status post-coronary artery bypass graft (CABG), ischemic cardiomyopathy, heart failure with reduced ejection fraction (HFrEF), paroxysmal atrial fibrillation on apixaban, chronic obstructive pulmonary disease, and a recent gastrointestinal bleed presented to our emergency department with new-onset aphasia and dysphagia, concerning for a cerebrovascular accident (CVA). NIHSS Stroke Scale was calculated at seven, as the scale does not account for aphemia, and the patient received three extra points on the mute/global aphasia section [6]. Initial computed tomography (CT) of the brain showed no evidence of intracranial hemorrhage or infarction (Figure 1), and a CT angiography of the intracranial vessels showed no acute thrombus (Figure 2). Given the time elapsed prior to the presentation, the patient was out of the window for thrombolytic use. Thereafter, magnetic resonance imaging (MRI) of the brain performed the following day showed a hyperdense lesion in the left frontal lobe on T2/FLAIR sequence and diffusion-weighted imaging (Figures 3, 4). Complete TTE was negative for an intracardiac thrombus, but severely reduced EF of 15-20% was noted with global hypokinesis. The physical exam was positive for aphemia rather than aphasia, as the patient was able to understand written language and write full sentences, but unable to perform the motor functions necessary for word production. No new gross motor or sensory deficits were found on extremity testing, nor were there any cerebellar deficits observed during the heel-to-shin test. After reviewing the infarct seen on the MRI, it was noted to be in the left precentral gyrus. The patient was evaluated by speech-language pathology and physical therapy, who provided goal-directed therapy for his disabilities. These therapies included proper bodily orientation while eating, taking smaller, more precise bites of food, and practicing swallowing different consistencies of liquids and solids in a controlled setting. Over the course of a few days, there was a notable improvement in his dysphagia, though he still had residual difficulty producing speech. Management thereafter focused on secondary stroke prevention, with aspirin and a high-intensity statin added to his medical therapy. His HFrEF therapy was reintroduced during his stay, including a beta blocker, angiotensin receptor blocker, mineralocorticoid receptor antagonist, and an SGLT2 inhibitor. He was later discharged home with home health and family support to continue recovering from his aphemia. The most recent follow-up clinic documentation from the Veterans Affairs clinic, approximately two months after discharge, noted that the patient had not regained complete speech production but was able to eat foods he previously enjoyed.

## Discussion

A thorough physical exam can serve as a physician’s primary tool to identify pathological mechanisms. When there is a concern for a stroke, a prompt and complete neurological examination is needed to isolate the location of the infarct. This is followed by imaging, with immediate CT scans of the brain to differentiate between hemorrhagic and ischemic etiologies. After appropriate therapy is provided for the underlying etiology of the cerebrovascular insult, examples include dual antiplatelet therapy (DAPT) for ischemic strokes [7] and blood pressure control or reversal of anticoagulation for hemorrhagic strokes [8], therapy focused on speech, mobility, and dexterity should be prioritized. Identifying key details in the physical exam can assist with early detection and localization of cerebral infarcts, ultimately helping guide the appropriate management. As aphemia is primarily a motor disorder, therapy should focus on restoring the function of the muscles involved in phonation and deglutition.

## Conclusions

Aphemia and aphasia are two disorders that are often difficult to distinguish. The clinical differentiator lies in the presentation of motor versus comprehension deficits. This case serves to highlight the difference between aphemia and aphasia and emphasize the importance of the physical exam as a valuable clinical tool in differentiating between the two. Additionally, it is important to distinguish between the two, as therapy differs and can focus on restoring the motor functions lost with aphemia. Our patient had fully intact language comprehension but exhibited a noticeable lack of the motor function of speech. As such, he was diagnosed with aphemia rather than aphasia. In such cases, following initial management that involves stabilization of the CVA, prompt initiation of physical, speech, and swallowing therapy can help recover the lost abilities associated with aphemia and provide patients with the skills to return to daily life with minimal disabilities.
